# Effects of Progesterone on Isoflurane Requirement for Balance Disturbance and Loss of Righting Reflex in Male Mice

**DOI:** 10.7759/cureus.76274

**Published:** 2024-12-23

**Authors:** Takeru Shimizu, Shoko Nakamura, Shinichi Inomata

**Affiliations:** 1 Department of Anesthesiology, University of Tsukuba, Tsukuba, JPN; 2 Department of Anesthesiology, University of Tsukuba Hospital, Tsukuba, JPN

**Keywords:** anesthetic requirement, balance disturbance, isoflurane, loss of righting reflex, progesterone, rolling response

## Abstract

Introduction

It has been known that progesterone has central effects, as measured by minimum alveolar concentration in various experimental settings. Previously, we showed that progesterone reduces the sevoflurane requirement for the loss of righting reflex (LORR) using male mice. However, the combination of progesterone and isoflurane has not been studied. Therefore, in this study, we compared the effect of progesterone on anesthetic requirements in a mouse model.

Methods

Male C57BL/6 mice were treated with either progesterone (75 mg/kg) + olive oil or only olive oil. Animals were studied in closed cylinders supplied with oxygen and isoflurane that rotated four times per minute. Balance disturbance and loss of the righting reflex were counted. The data were analyzed by using a multiple independent variable logistics regression model.

Results

The concentrations at the onset of balance disturbances, represented by the effective dose 50% (ED_50_) and effective dose 95% (ED_95_) of isoflurane, were 0.37% and 0.45% for the control group and 0.34% and 0.41% for the progesterone group, respectively. Similarly, the concentrations for loss of righting reflex (LORR), represented by ED_50_ and ED_95_, were 0.55% and 0.62% for the control group and 0.53% and 0.60% for the progesterone group, respectively. Subcutaneous injection of progesterone at a dose of 75 mg/kg significantly reduced the isoflurane requirement for both balance disturbance (p = 0.0022) and LORR (p = 0.0218).

Conclusion

We conclude that progesterone decreased isoflurane concentration for both balance disturbance and LORR.

## Introduction

Progesterone is known as a strong positive allosteric activator of γ-aminobutyric acid type A (GABAA) receptors and to work against central nervous system functions [1]. Those include phenomena similar to sedatives and anesthetics [2-4]. Although several studies have been done to compare the anesthetic requirements by using minimum alveolar concentration (MAC) [5-7] and bispectral index (BIS) [8], results of the studies varied, demonstrating decreased MAC [5] or no such phenomenon even with higher progesterone levels [6,7]. So far, we reported that sevoflurane requirement for the loss of righting reflex (LORR) had been reduced by the exogenous progesterone [9]. However, the effect of exogenously increased progesterone has not been examined with isoflurane. Although it seems that the proportion of isoflurane usage as volatile anesthetics has been declining, isoflurane is still significantly used worldwide [10,11]. The ratios of involvement of GABAA receptors are similar in sevoflurane and isoflurane, but those of other receptors including glycine are different [12,13]. It is yet unknown whether exogenous progesterone may change isoflurane requirement as much as that observed in sevoflurane.

Therefore, the aim of this study was to examine the isoflurane requirements not only for LORR but for balance disturbance in male mice with and without progesterone. We tested the hypothesis that increased progesterone levels is associated with lower concentrations required for balance disturbance and LORR.

This article was previously presented as a meeting abstract at the 2018 ASA Annual Meeting in San Francisco on October 14, 2018. This article was previously posted to the Research Square preprint server on October 9, 2023.

## Materials and methods

Animals

All animal procedures were approved by the University of Tsukuba Animal Care and Use Committee (16-116). C57BL/6 mice (male, six to 10 weeks old, weight 25-32 g) were obtained from Charles River Laboratories Japan (Yokohama, Japan). The experiments were done in compliance with ARRIVE (Animal Research: Reporting of In Vivo Experiments) guidelines. No more than five mice were housed in a cage. Animals were kept in a controlled environment (lights on 8:00 AM-8:00 PM; temperature: 20-22°C) with ad libitum access to food and water.

Drugs and groups

Animals were divided into two groups: the progesterone group and the control group. Computer-generated random number tables were used to allocate the animals. In the progesterone group, subjects were received subcutaneously in the scruff of the neck progesterone (Sigma-Aldrich, St. Louis, MO) 75 mg/kg dispersed in 0.1 mL of olive oil (Sigma-Aldrich, St. Louis, MO). In the control group, subjects received 0.1 mL of only olive oil (vehicle) subcutaneously. Injection was done one hour before each set of experiments.

Apparatus

Mice were assigned rolling responses, as reported by Robbins, to assess the anesthetic end-point [14]. A rotational cylinder (internal diameter 14 cm) made of steel mesh was built in a 1.8-liter chamber. Five animals from the same cage were contained in the mesh cylinder, and the mesh cylinder was set in the chamber to be rotated. Then, oxygen (0.5 L/minute) and isoflurane were supplied to the airtight chamber. Isoflurane concentration was increased by 0.05% intervals under 1.0% and 0.1% over 1.0% by using an anesthetic vaporizer (Penlon PPVΣ, Penlon, Abingdon, UK) until all mice lost the righting reflexes. A multi-gas analyzer (Capnomac Ultima, Datex, Helsinki, Finland) was used to measure the concentrations inside the chamber by connecting the sampling tube to the sampling port of the chamber around the height of the rotating cylinder. The first trial of each designated concentration was done at least 10 minutes after maintaining the target value.

Assessment of anesthetic sensitivity

The cylinder was rotated at the rate of 4 rev/minute. The state in which animals were unable to maintain an upright position and rolled over in the cylinder during any revolution of the chamber but were able to restore all of their paws to the floor of the chamber was defined as balance disturbance. The state in which animals were placed on their back completely and continuously for 15 seconds was defined as the LORR. Allocation of the drugs was concealed until injection for each experiment. Animal responses were observed by experimenters who were blinded to allocation. Two observers attended each experiment. Statistical analysis was done by experimenters who were also blinded to allocation. Only incremental measurements were done in this study. All experiments were done between 9:00 AM and 5:00 PM. Twelve independent experiments were carried out using five animals at a time. Animals were marked so that they could be identified individually. Each measurement for a certain concentration of isoflurane consisted of three sets. Five rotations were applied in each set, and any change in any set of the series was counted as either balance disturbance or LORR. We allowed five minutes for each set of the certain concentration. Rotational speed was all the same.

Body temperature measurements

We conducted a separate experiment to measure tympanic temperatures using an electrical thermometer (Data Logger Thermocouple Thermometer, Muromachi Kikai Co., Ltd., Tokyo, Japan) just after induction of anesthesia and 3.5 hours after induction. Male mice of similar age and size were used. Animals were placed in the same apparatus and anesthetized with isoflurane in oxygen at 0.5 L/minute during the experimental period. The cylinder was not rotated.

Statistical analysis

Isoflurane concentration to be reduced was analyzed by a multiple independent variable logistic regression model using SAS System for Windows, version 6.12 (SAS Institute Inc., Cary, NC) that included an interaction term for isoflurane concentration and progesterone dose. Whether isoflurane and progesterone independently affected the balance disturbance was determined as the main effects components. Whether isoflurane and progesterone interacted to affect LORR was determined as the interaction coefficient. The maximum likelihood ratio test was performed to determine which of the independent variables influenced the model. For determination of the concentration of isoflurane that makes 50% of mice stumbled (balance disturbance) or complete rollover (LORR), the probability of causing balance disturbance or LORR was set to the value of 0.5, and the equations were solved for the isoflurane concentrations.

In the same manner, for determination of the concentration of isoflurane that causes balance disturbance or LORR in 5% or 95% of mice (95% confidence interval [CI]), the probability to cause balance disturbance or LORR was assigned at 0.05 or 0.95, and the isoflurane concentrations were calculated.

We calculated the sample size based on our pilot study using G*Power 3 software (Heinrich-Heine-Universität Düsseldorf, Düsseldorf, Germany). A one-tailed test with a power of 90% and a type I error of 5% (α = 0.05 and β = 0.10) gave the minimum sample size of 28 animals for each group to detect a 0.8 effect size. Two animals were added just in case compensation was needed.

For statistical comparisons, a chi-square test between these groups was performed (StatView software, SAS Institute Inc., NC). p-values < 0.05 were considered to be significant.

## Results

Twelve independent experiments were carried out using five animals at a time. The data were obtained from 30 animals for each group, 60 animals in total. No animals were excluded. Numbers and percentages of mice that had shown balance disturbance and LORR were shown in Table 1.

**Table 1:** Concentrations of isoflurane and number of animals observed as balance disturbance and LORR Each set of experiments included five animals at a time. Six sets of independent experiments were done. Therefore, 30 animals were included in each designated concentration of isoflurane. LORR: loss of righting reflex

Investigated isoflurane concentration (%)	Balance disturbance		LORR	
	Control n (%)	Progesterone n (%)	Control n (%)	Progesterone n (%)
0.00	0 (0)	0 (0)	0 (0)	0 (0)
0.10	0 (0)	0 (0)	0 (0)	0 (0)
0.20	0 (0)	0 (0)	0 (0)	0 (0)
0.25	1 (3.3)	1 (3.3)	0 (0)	0 (0)
0.30	3 (10)	5 (17)	0 (0)	0 (0)
0.35	12 (40)	16 (53)	0 (0)	0 (0)
0.40	23 (77)	28 (93)	0 (0)	0 (0)
0.45	28 (93)	30 (100)	2 (6.7)	2 (6.7)
0.50	30 (100)	30 (100)	7 (23)	9 (30)
0.55	30 (100)	30 (100)	17 (57)	21 (70)
0.60	30 (100)	30 (100)	28 (93)	28 (93)
0.65	30 (100)	30 (100)	30 (100)	30 (100)

**Figure 1:**
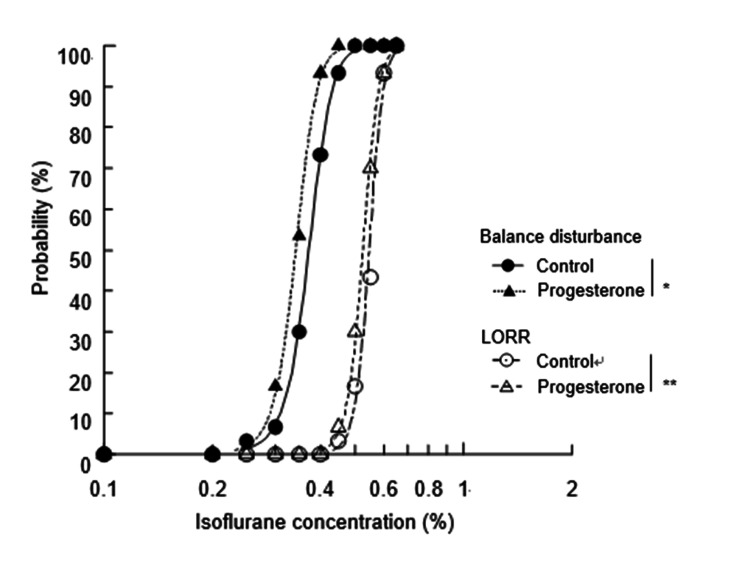
Isoflurane concentrations calculated by logistic regression for balance disturbance and LORR Isoflurane concentrations versus probability to stumble (balance disturbance)/complete roll (LORR) are shown. Administration of progesterone significantly reduced isoflurane requirement for both balance disturbance (*p = 0.0022) and LORR (**p = 0.0218).

Figure 1 shows isoflurane concentrations calculated by logistic regression.

Concentrations of isoflurane for the onset of balance disturbances are as follows. For the control group, the effective dose of 50% (ED_50_) was 0.37% (95% CI: 0.36-0.39), and the effective dose of 95% (ED_95_) was 0.45% (95% CI: 0.43-0.49). For the progesterone group, the ED_50_ was 0.34% (95% CI: 0.33-0.37), and ED_95_ was 0.41% (95% CI: 0.39-0.46). Concentrations for LORR are as follows. For the control group, the ED_50_ was 0.55% (95% CI: 0.53-0.56), and the ED_95_ was 0.62% (95% CI: 0.60-0.65). For the progesterone group, the ED_50_ was 0.53% (95% CI: 0.51-0.54), and ED_95_ was 0.60% (95% CI: 0.58-0.64) (Table 2).

**Table 2:** Isoflurane concentrations required for balance disturbance and LORR ED_50_: effective dose 50%; ED_95_: effective dose 95%; LORR: loss of righting reflex; Prog: progesterone

Assessment	Prog	ED_50_ (%)	CI	ED_95_ (%)	CI
Balance disturbance	-	0.37	0.36-0.39	0.45	0.43-0.49
	+	0.34	0.33-0.35	0.41	0.39-0.44
LORR	-	0.55	0.53-0.56	0.62	0.60-0.65
	+	0.53	0.51-0.54	0.60	0.58-0.64

For body temperature measurements, seven male animals were used. The body temperatures just after induction and after 3.5 hours were 35.0 ± 1.4°C and 33.7 ± 0.6°C, respectively, and there was no significance (p = 0.09).

Subcutaneously injected progesterone 75 mg/kg significantly reduced isoflurane requirement for both balance disturbance (p = 0.0022) and LORR (p = 0.0218).

## Discussion

This study demonstrated that subcutaneous injection of progesterone 75 mg/kg reduced isoflurane concentration significantly for both balance disturbance and LORR in male mice with regard to rolling response. These results are consistent with our previous study, which found that exogenous progesterone reduced sevoflurane requirement for LORR in male mice [9]. Several phenomena that also support the results have been published previously, including a decreased requirement for inhaled anesthetics during pregnancy [15], decreased MAC during early pregnancy [16] and in the period just after delivery [17], and narcotic effects [18,19]. Reddy et al. showed that a higher dose of progesterone treatment (50 and 75 mg/kg) had been effective in reducing anxiety but not a lower dose (10 mg/kg) [20]. Likewise, our previous study showed that the dose of 75 mg/kg, but not 37.5 mg/kg, had decreased sevoflurane concentration. Therefore, we tested the dose of 75 mg/kg for these experiments and found evidence that exogenous progesterone affected the isoflurane requirement.

The rolling response was focused on our experimental design, in which balance impairment was regarded. The current data revealed that progesterone affected the balance control at comparatively low isoflurane concentration. The state in which animals are tripped is connected with the GABA-mediated pontine reticular formation [21], as well as vestibular disturbances [22]. The current study revealed that animals showed balance disturbance at the isoflurane concentration as low as 0.34% in the condition of the higher progesterone level. Consequently, we can postulate that females in the luteal phase are susceptible to balance disturbances, which may increase the probability of balance disturbances. Therefore, medical staff must keep an eye on these patients, especially in case of day surgeries. As another clinical feature, a disparity in the degree of LORR during anesthesia could occur. Female patients with higher progesterone levels could be paralyzed at remarkably low MAC of anesthetics [21], which may cause adverse events, including intraoperative awareness or adverse phenomena such that patients are paralyzed with consciousness. This speculation can be reinforced by previous findings [23]. These events would be obvious when comparing the groups not only between males and females but also between females in the different phases of the menstrual period. In ordinary clinical settings, other drugs are often used for both induction and maintenance of anesthesia, including intravenous anesthetics, opioids, and muscle relaxants. It has been reported that the administration of muscle relaxant cisatracurium significantly reduced the requirement of both sevoflurane and propofol [24] and that the administration of opioids reduced the isoflurane requirement in animal models [25]. Thus, even if the difference in isoflurane requirement by progesterone is small, the interaction of other drugs, especially continuous infusion of opioids for postoperative analgesia, may impact the outcomes. Medical staff must be aware of these aspects. 

The differences in the concentrations required for LORR with or without progesterone between sevoflurane from our previous study and isoflurane from the current study were compared. The reported MAC for isoflurane and sevoflurane values were used (1.85% for isoflurane and 3.25% for sevoflurane) [26]. Then, only a 1.1% difference made the statistical difference in isoflurane, whereas a 3.1% difference was required in sevoflurane. These differences could be caused by the differences in the involvement of molecular targets in the effects of isoflurane and sevoflurane. It has been reported that the effects of isoflurane were mediated by 36% by GABAA receptors and 39% by glycine receptors [12], whereas the effects of sevoflurane were mediated by 38% by GABAA receptors and 45% by glycine receptors [13]. This means that the effects of isoflurane are mediated by as much as 25% of other receptors, whereas only 17% could mediate in sevoflurane. Further work is required to elucidate the precise mechanism.

The estimated time of the time constant for this apparatus is about 11 minutes. For the commencement of each trial of the designated concentration, we kept it for at least 10 minutes. It required several extra minutes to confirm the evenness of isoflurane in the chamber. We carried out three sets of five revolutions at an interval of five minutes. Therefore, the last set at a certain concentration was done at least 24 minutes after equilibrium had been achieved. The pharmacokinetics, as defined as the ratio of end-tidal anesthetic concentration to inspired anesthetic concentration of isoflurane, showed that the values after 10 minutes and that after 25 minutes were already similar and near plateau [27].

Body temperature is the confounding factor that can affect the anesthetic requirement. We did not measure the body temperature during the experiments in order to avoid disturbing the intra-chamber condition by opening it to the atmosphere. At the same time, non-contact thermometers cannot be used through the resin chamber wall, or subcutaneous implantable devices can be invasive and less commonly available [28]. Since each experiment required 3 to 3.5 hours, we kept the mice anesthetized for 3.5 hours. The body temperature change was -1.3 ± 1.8°C, and this would be negligible [29].

Our study has several limitations. Firstly, the plasma levels of progesterone were not measured. Therefore, it is unclear whether the progesterone was high enough to trigger the associated receptors. Secondly, the current experimental system determines a spinally determined anesthetic phenotype, including balance disturbance and LORR. The effects of progesterone vary according to the measures, leaving future investigations. Thirdly, we did not examine the effect of exogenous progesterone in female animals. Therefore, it is yet unclear whether additional progesterone can exert further anesthetic actions. However, female animals have intrinsic estrogen, which exerts the opposing effect by suppressing GABAA receptors [30]. Thus, male animals are suitable in the sense that the effect of GABAA activation by progesterone should be examined. Finally, we did not examine other doses of progesterone, as compared to our previous study. Further experiments with ample samples are required to elucidate differences.

## Conclusions

The current study showed that exogenous progesterone (75 mg/kg) reduced the isoflurane requirement for balance disturbance as well as LORR. This finding illuminated that progesterone affects anesthetic requirement with isoflurane as well as sevoflurane. Our results may suggest the necessity of adjustment for the anesthetic requirements in sex differences in clinical settings.
